# Linking *S. aureus* Immune Evasion Mechanisms to Staphylococcal Vaccine Failures

**DOI:** 10.3390/antibiotics13050410

**Published:** 2024-04-30

**Authors:** Irshad Ahmed Hajam, George Y. Liu

**Affiliations:** 1 Department of Pediatrics, University of California San Diego, San Diego, CA 92093, USA; ihajam@health.ucsd.edu; 2 Division of Infectious Diseases, Rady Children’s Hospital, San Diego, CA 92123, USA

**Keywords:** *S. aureus*, MRSA, vaccines, immune imprinting, immune evasion, vaccine interference

## Abstract

Vaccination arguably remains the only long-term strategy to limit the spread of *S. aureus* infections and its related antibiotic resistance. To date, however, all staphylococcal vaccines tested in clinical trials have failed. In this review, we propose that the failure of *S. aureus* vaccines is intricately linked to prior host exposure to *S. aureus* and the pathogen’s capacity to evade adaptive immune defenses. We suggest that non-protective immune imprints created by previous exposure to *S. aureus* are preferentially recalled by SA vaccines, and IL-10 induced by S. aureus plays a unique role in shaping these non-protective anti-staphylococcal immune responses. We discuss how *S. aureus* modifies the host immune landscape, which thereby necessitates alternative approaches to develop successful staphylococcal vaccines.

## 1. Introduction

*Staphylococcus aureus* (SA) is a highly adaptable pathobiont that colonizes the skin and mucosal surfaces in approximately 30% of individuals [1,2]. SA’s ability to survive in diverse tissues enables the infection of the skin and soft tissues, pneumonia, endocarditis, osteomyelitis, and bacteremia. SA is a leading cause of infections within healthcare facilities in the United States, with approximately 20,000 individuals succumbing to invasive diseases in 2017 [3]. After the introduction of antibiotics targeting SA, resistance has emerged against nearly all antibiotics. A recent report on antimicrobial resistance (AMR) in 2016, commissioned by the UK government, projected that, if no action is taken to tackle the AMR imposed by ESKAPE pathogens (Enterococcus, Klebsiella, SA, Acinetobacter, Pseudomonas, and Enterobacter), ten million attributable deaths could occur annually by 2050 [4]. Because of the sluggish progress in the development of novel antibiotics and the swift emergence of AMR, vaccination represents a needed complementary approach to address infections caused by these pathogens. Remarkably, there is still no vaccine available against these organisms, despite abundant attempts.

SA has been the target of vaccine development since 1902 [5]. Although pre-clinical research has yielded a broad array of seemingly effective vaccines targeting toxins and cell wall-anchored (CWA) antigens [6,7], remarkably, none of these vaccines have proven effective after approximately thirty clinical trials [8,9]. As a successful pathobiont, SA has evolved a large repertoire of immune evasive strategies that serve to maintain its coexistence with the human host in colonization [10,11,12] or infections [13,14]. These tactics affect both adaptive branches of the immune system, and certainly affect the efficacy of SA vaccination. However, it has not been clear how any particular immune evasion mechanism is linked to the failure of SA vaccines in the clinical trials. To date, the majority of vaccines have been evaluated in naive animal models, in contrast to humans who frequently encounter SA from early infancy [1,2]. Investigating animal models pre-exposed to SA has opened up a broader appreciation of the complex biology underlying the interaction between the vaccine and the immune system that has been modified by the pathobiont. Insights gained from these studies critically point to novel approaches to vaccination that could overcome current staphylococcal vaccine challenges. In this review, we explore key principles in the intricate interactions between SA and its host, and discuss how the previous exposure to SA affects vaccine efficacy, including the critical role of immunosuppressive cytokine IL-10. We discuss newly found vaccines strategies aimed at overcoming SA interference with vaccination.

## 2. How SA Evades Host Adaptive Immune Defense

SA produces a plethora of virulence factors that promote pathogen survival in diverse host environments, thereby facilitating both infections and colonization [15]. Protein A (SpA), an extensively studied surface protein of SA, counters immune responses primarily by interacting with the antibody constant domain (Fc), hindering the phagocytosis of SA and impairing bacterial elimination [16,17]. Additionally, SpA binds the heavy-chain variable region of the B-cell receptor (BCR), initiating supra-clonal expansion and the subsequent apoptosis of B1 and marginal zone (MZ) B cells [18,19], thereby dampening the humoral response against staphylococcal antigens. Moreover, SpA cross-links BCRs to activate and promote the generation of IL-10-producing immunosuppressive B cells [20]. This mechanism strengthens SA’s ability to evade humoral immune responses. Consistent with the immunosuppressive nature of SpA, vaccination with a mutant form of SpA (SpA_KKAA_) lacking the ability to bind either Fcγ receptor or Fab V_H_3 resulted in a heightened opsonophagocytic clearance. Furthermore, SpA_KKAA_ vaccination elicited a more robust and diverse antibody (Ab) response against SA antigens in mice [21]. A similar virulence factor, Staphylococcal immunoglobulin-binding protein (Sbi), which shares the IgG-binding function of SpA, further expands SA’s capacity to evade antibody-mediated clearance [22,23].

In addition to SpA, various SA-secreted toxins, such as LukED and hlgACB, impede anti-SA Ab responses, indicating their potential involvement in modulating humoral immunity [24].

It is notable that the antibody engagement of complement and myeloid cells drives the effective killing of pathogens. SA is well adapted to evade macrophage and neutrophil antimicrobial functions, including reactive oxygen species, antimicrobial peptides, and neutrophil extracellular traps [25,26]. Hence, the presence of protective antibodies alone is insufficient to assure the optimal clearance of SA. Overall, it is apparent that SA has multiple strategies to undermine the humoral component of the host’s adaptive immune system, which likely impact the effectiveness of anti-SA vaccination.

It is notable that individuals with B-cell deficiency are generally not at higher risk of infection with SA compared to normal individuals [27]. In comparison, T cells have a more pronounced effect on limiting SA infections, as evidenced by studies in both humans and experimental mouse models [28,29]. Robust evidence underscores the pivotal contribution of CD4^+^ T cells, particularly Th1 and Th17 cells, in orchestrating protection against SA infections [30,31,32]. Given the critical role of T-cell immunity in conferring protection, SA has developed several mechanisms to subvert host T-cell defense. SA employs strategies that primarily target the effector functions of T cells, facilitated by two major classes of virulence factors: superantigens and secreted toxins [12,33,34]. Superantigens such as Toxic Shock Syndrome Toxin-1 (TSST-1) and Enterotoxin B (SEB) induce nonspecific and potent activation of up to twenty percent of peripheral T cells [34]. This indiscriminate activation disrupts the focused and coordinated anti-SA immunity, and consequently, leads to a reduced overall T-cell receptor diversity and impaired development of robust antigen-specific protective T-cell responses. Other than superantigens, secreted toxins such as leukotoxins, hemolysins, and phenol soluble modulins (PSMs) can undermine protective T-cell responses through several other mechanisms [35,36,37,38]. These toxins may directly limit Th1/Th17 cells or the dendritic cell (DC) orchestration of antigen-specific T-cell development. Moreover, PSMs can promote the generation of IL-10-producing tolerogenic DCs, fostering immunosuppressive regulatory T cells (Tregs), which diminish IL-10-dependent effector T-cell responses during a persistent infection [36,39,40]. Consistent with these mechanisms, T cells from a primary SA infection fail to confer robust secondary protection. Lee et al. indicated that a primary SA infection compromised the development of memory T-cell responses through the depletion of local DCs mediated by α-toxin [41].

T-cell activity is tightly regulated by checkpoint inhibitors, which pathogens could exploit as part of their survival strategy within the host. Several clinical isolates of SA have been shown to directly interact with PD-1 to inhibit T-cell activation [42]. This strategy of suppressing T-cell activity is not unique to SA, as other pathogens, including *Helicobacter pylori*, *Mycobacterium tuberculosis*, *Listeria monocytogenes*, and *Escherichia coli*, are also capable of manipulating the PD-1 pathway to circumvent the host’s immune defenses [43,44,45].

Another tactic employed by SA involves the molecular mimicry of host immune components. SA secretes a class II MHC analog protein, MAP, which disrupts T-cell proliferative responses and promotes Th2 cell differentiation [46]. This is significant as Th2-associated cytokines, like IL-4 and IL-10, can dampen the IL-17 response [47,48]. SA also induces myeloid-derived suppressor cells (MDSCs) during a chronic infection, which are known to suppress T-cell responses in an IL-10-dependent manner [49]. In addition to manipulating MDSCs, SA also directly stimulates monocytes/macrophages and regulatory B1a cells to bypass host immune defenses through TLR2-IL-10-dependent pathways [50,51,52].

Although SA soft tissue infections are common, a longstanding SA colonization likely plays a greater role in the modulation of host immune responses and vaccines. SA colonizes and infects humans from early infancy, with as many as fifty percent of infants having encountered SA by the age of 6 months [1]. A study by Kelly et al. demonstrated that IL-10 plays a critical role in aiding SA to establish its niche within the nasal cavity [53]. This body of research strongly supports the notion that SA-induced IL-10 production is a principal mechanism by which SA undermines host immunity to enhance its survival within the host. The significance of IL-10 in the immune response to SA will be explored in depth in the subsequent sections.

## 3. Significance of IL-10 in SA Immunity

The immune system has developed complex mechanisms to combat infections while mitigating tissue damage. Several regulatory pathways help to strike a delicate balance between a robust anti-pathogen response and the prevention of excessive tissue pathology. Among these mechanisms, the cytokine IL-10 stands out as a pivotal player in regulating inflammation [54,55]. IL-10 serves as a potent anti-inflammatory cytokine, crucial for shielding the host from overly aggressive immune responses to pathogens partially by controlling effector T-cell responses [55]. Although IL-10 protects the host against overtly inflammatory tissue damage, certain bacteria exploit this immunosuppressive mechanism to facilitate their persistence within the host. For example, *M. tuberculosis* [56,57] and *Bordetella pertussis* [58,59] enhance their survival in the host by inducing the production of IL-10 from innate immune cells to suppress Th1-type immunity. Furthermore, the cells that produce IL-10 directly drive naïve T cells towards a regulatory phenotype, further weakening the protective T-cell responses [60]. Thus, IL-10 acts as a double-edged sword: on the one hand, it protects the host against excessive inflammatory responses, but on the other, it aids the pathogen in preserving its niche within the host.

SA adeptly manipulates the host IL-10 responses, leveraging this capability as a key strategy for immune evasion during both chronic and biofilm-associated infections [52,61]. The role of IL-10 in fostering a SA–host relationship is well-documented in both the clinical and mouse literature on SA colonization and infection dynamics. During colonization, IL-10 produced by macrophages enable the SA’s colonization of the nasal passage via dampening local T-cell-mediated immune responses [53]. IL-10, secreted by MDSCs, also supports the SA biofilm through a histone deacetylase complex-dependent mechanism [61]. In the setting of active infection, the role of IL-10—produced abundantly by various cell types—varies, offering either protection or harm to the host depending on the site of SA infection [52].

In acute systemic SA infections, IL-10 serves an indispensable and unique role, distinct from other regulatory pathways, by safeguarding the host from severe immunopathology and bacterial dissemination through the modulation of both local and systemic inflammatory responses [52]. In the absence of IL-10, an upsurge of IFN-γ and IL-17 from T cells can causing enhanced neutrophil migration to the infection site and increase host mortality [62,63].

In contrast to acute systemic infections, where IL-10 production typically benefits the host by mitigating against excessive inflammation and immunopathology, IL-10 facilitates SA persistence role during acute localized skin infection. The SA infection of IL-10-deficient mice demonstrated that a low IL-10 concentration results in diminished skin lesions and reduced bacterial burden [52]. This difference of IL-10′s impact on skin and systemic infections may be partly linked to differences in the primary cellular sources of IL-10 in various infection contexts. Specifically, in localized skin infections, macrophages and MDSCs emerge as the principal sources of IL-10. Conversely, in the setting of acute systemic infections, CD19^+^CD11b^+^CD5^+^ B1a cells were identified as the predominant producers of IL-10 [52]. Notably, in an adoptive transfer model, B1a cells confer protection to IL-10-deficient mice against systemic SA infection, underscoring the complex and context-dependent roles of IL-10 in modulating host responses to SA infections.

In addition to the role of IL-10 during infections, IL-10 play a critical role in establishing SA nasal colonization [53]. Understanding the intricate mechanisms through which SA colonizes the nasal passages is crucial not only for developing novel approaches to eradicate SA colonization but also for understanding the downstream impact of nasal colonization on infection dynamics and the feasibility of vaccination strategies. While the relationship between Abs and SA nasal colonization remains unclear, T cells, particularly those involved in the Th17 response, are crucial in limiting SA presence in the nasal passages [53]. A study by Kelly et al. shows that IL-10 dampened protective local inflammatory Th17 cellular responses, resulting in an inefficient clearance of bacteria from the nasal cavity [53]. Studies in IL-17-deficient mice showed a higher bacterial load during SA nasal colonization. This mechanism aligns with observations in human populations, in which HIV-infected individuals, who experience significant depletion of Th17 cells, show a higher degree of SA colonization and skin and soft-tissue infections [64,65,66,67]. Furthermore, a study cohort consisting of SA nasal carriers and non-carriers demonstrated that carriers produce higher levels of IL-10 in response to heat-killed SA, whereas the production of TNF-α remained consistent between the two groups [68]. This increase in IL-10 production among carriers could play a crucial role in facilitating the commensal relationship with SA, suggesting a potential mechanism by which SA manages to evade host immune responses and establish itself as a part of the normal flora in some individuals.

Research into the bacterial factors that drive IL-10 production could offer insights crucial for designing effective preventive measures against SA infections. A report showed that SA peptidoglycan directly activates human innate immune cells, leading to a robust IL-10 response mediated by the TLR2 pathway [50]. Additionally, we demonstrated that the O-acetylation of cell wall peptidoglycan suppresses pro-inflammatory cytokines, such as IL-1β, IL-6, IL-22, and TGF-β, while enhancing IL-10 production, thereby modulating Th17 development [48]. Other virulence factors, such as PSMs and SpA, also contribute to IL-10 production by inducing tolerogenic DCs, which promote regulatory Treg differentiation and subsequent immune suppression [39,69]. Notably, a study by Heim et al. revealed that SA-induced lactate production triggers an IL-10 response independent of TLR2 and involving metabolites as an alternative pathway in triggering IL-10 production from host cells [61]. These findings underscore the complexity of SA–host interactions, with IL-10 serving as a critical mechanism by which SA evades host defenses and establishes colonization and chronic infections.

## 4. Effects of SA Exposure and IL-10 on Staphylococcal Vaccination

Humans routinely encounter SA early on in life and leave behind durable SA antigen-specific immune memory imprints [2,70,71]. Numerous investigations have assessed for the presence and functions of serum anti-SA Abs following human exposure to SA [72,73]. These studies have consistently reported strong Ab responses directed against SA antigens, including CWA proteins and toxins. While Ab responses to CWA antigens appear to have a limited protective capacity [10,71,73,74], Abs targeting toxins are suggested to be more effective against SA infections [71]. The exact impact of these Abs on vaccine efficacy in humans remains to be fully understood. However, the failures of apparently promising vaccines in clinical trials, despite efficacy in preclinical studies, point to a possible link between pre-existing immunity and the failed SA vaccines. This has led us to consider if immune imprinting (original antigenic sin hypothesis or OAS) plays a role in SA vaccine failures. OAS proposes that the immune system’s memory response to an initial influenza infection is preferentially recalled upon host infection by a related influenza strain or vaccine, which can lead to the loss of protective immune response against the virus [75]. Similarly, non-protective immune memory established by either SA colonization or infection could be significantly recalled, thereby influencing the effectiveness of vaccines.

To test this hypothesis, we simulated the unsuccessful human vaccine trial that targeted the staphylococcal iron transporter IsdB [76]. We first showed that mice previously infected with SA harbor non-protective IsdB antibody imprints [10] that were non-neutralizing and that showed hyper-sialylation that interfered with the opsonophagocytic killing of SA by neutrophils. The non-protective IsdB-specific B-cell response was preferentially recalled by IsdB vaccination in SA pre-exposed mice, which led to ineffective vaccination. In addition, the non-protective IsdB Abs diminished the effectiveness of protective IsdB-specific Abs by direct competition. In support of this hypothesized antibody competition mechanism, we showed that purified human anti-SA Abs from healthy volunteers reduced the efficacy of two anti-SA monoclonal Abs, anti-αToxin Suvratoxumab [77] and anti-Clumping Factor A Tefibazumab [78], which were unsuccessfully used in clinical trials [71]. Thus, the observed mechanism of antibody interference in our murine model could explain the lack of success associated with both active and passive SA immunization trials.

For T cells, the work by Hendriks et al. underscored the presence of SA-specific T-cell imprints in healthy individuals, potentially derived from colonization and infection [70]. Montgomery and colleagues showed that T-cell imprints induced to SA toxins after staphylococcal infection are moderately protective and reduced the efficacy of highly effective SA toxin vaccines [79]. In contrast to toxin imprints, we showed that SA infection induces a non-protective anti-CWA T-cell memory response, which, upon CWA vaccination, are recalled to drive a non-protective vaccine response [80]. Hence, immune imprinting can negatively impact T-cell as well as B-cell vaccine responses.

**Figure 1:**
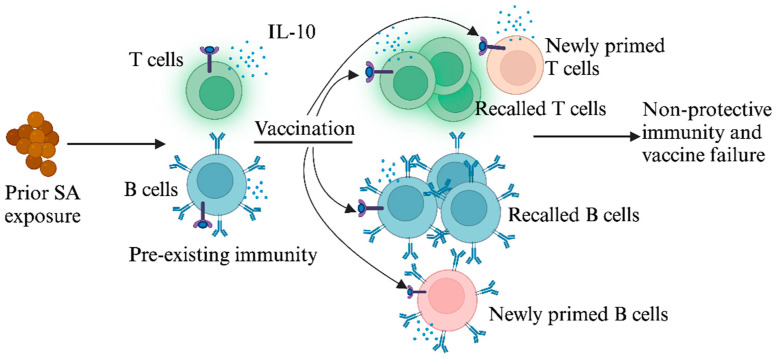
**Prior exposure to SA dampens protective responses to SA vaccination.** Initial exposure to SA induces T and B cells that produce the immunosuppressive cytokine IL-10. When subsequently vaccinated with SA antigens, these pre-existing T and B cells are preferentially recalled, resulting in ineffective vaccine responses.

While non-protective immune imprints predicted the outcome of failed vaccination in mice and provided a plausible explanation for the failure of SA vaccine trials, it has remained unclear why CWA immune imprints are non-protective in the first place. In the case of influenza, imprints were protective against the original strain of influenza. For SA, we recently reported that IL-10 may be a central driver of non-protective imprints [80]. IL-10, which is abundantly secreted in association with prior SA infection and colonization, directly dampens IsdB vaccine efficacy [48,52,53] (Figure 1). Although innate immune cells are major producers of IL-10 during acute and chronic infections, we showed that IL-10 secretion by CD4^+^ T cells is critical for blunting protective Th17 vaccine responses [80]. This finding aligns with our earlier work, which more precisely defined the role of SA-induced IL-10 in the dampening of host Th17 protective responses against SA [48]. The role of IL-10 in undermining vaccine efficacy extends beyond SA to other pathogens as well. Research by Pitt et al. demonstrated that an IL-10-rich environment impeded the development of optimal IFN-γ and IL-17A T-cell responses after Bacillus Calmette–Guerin (BCG) vaccination [57]. Furthermore, T cells primed in an IL-10-dominant setting exhibit persistent functional incompetence even after transference into low-IL-10 environments and failed to protect the host against *M. tuberculosis* [81]. These findings have clear therapeutic implications that are further discussed below.

Although the modulatory effect of CD4^+^ Th17 by IL-10 is well defined, it is not clear if and how IL-10 contributes to non-protective anti-SA Ab function. We have shown that the hyper-sialylation of IsdB-specific antibodies directly contributes to the loss of opsonophagocytic function [10]. IL-10 has been shown to regulate the glycosylation of T cells to decrease CD8^+^ T-cell sensitivity to antigens in response to a viral pathogen [82], and various cytokines have been shown to directly influence antibody glycosylation [83,84]. Thus, it would be worth investigating if IL-10 is linked to the hyper-sialylation of non-protective anti-SA antibodies.

In addition to immune imprinting, SA harbors several virulence mechanisms that could negatively impact vaccine-induced B- and T-cell responses. In a murine model of SA reinfection, Keener et al. demonstrated that SpA altered the fate of plasma cells by enhancing short-lived extrafollicular plasma cell responses, while simultaneously reducing the pool of long-lived plasma cells [85]. SA-secreted LukED toxin specifically targets and eliminates the predominant CCR5-positive effector memory T cells [38], suggesting the potential for SA to alter vaccine efficacy. SA can directly modulate T-cell responses via the PD-1 receptor, a suppression mechanism that has been demonstrated to be reversible by the application of anti-PD-1 Abs [42], suggesting potential avenues for mitigating SA’s immune evasion tactics.

## 5. Development of a Staphylococcal Vaccine That Overcomes Immune Imprinting

**Figure 2:**
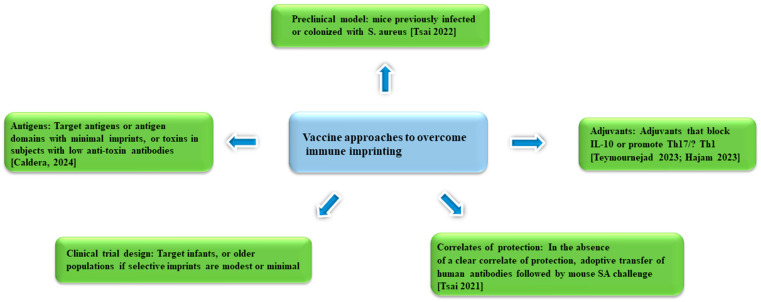
Approaches to SA vaccine development that aim to overcome immune imprinting [10,71,79,80,92].

The persistent failure in staphylococcal vaccines has long been a perplexing challenge that is proposed to stem from the suboptimal choice of staphylococcal antigens, adjuvants, pre-clinical model, and clinical trial design. This section will examine each of these factors while offering new perspectives from the perspective of immune imprinting (Figure 2).

Above, we discussed how a model that considers prior SA exposure could more readily explain the failure of SA vaccines. Humans are routinely exposed to SA, which leads to a build-up of robust anti-SA immune memory from an early age [1]. We have demonstrated that the highly effective IsdB vaccine becomes ineffective in mice previously exposed to SA [10], underscoring the limitations of the naïve mouse models mimicking human conditions. Although we have modeled prior exposure with a series of systemic SA infections, it is not clear what would be an optimal animal model that mimics humans pre-exposed to SA. Since humans are frequently colonized and occasionally infected with SA, both events likely contribute to imprints that shape the efficacy of subsequent SA vaccination. Whether SA colonization and infection induce subtle differences in non-protective imprints is not clear. However, our current model of SA pre-exposure, consisting of repeat systemic infections, generates antibody imprints that appear to be functionally similar to anti-SA imprints isolated from healthy human subjects [71]. Other models exist that could further refine the modeling of human conditions for the interrogation of SA vaccine efficacy. For example, studies have revealed that laboratory mice with a “wild” microbiome exhibit immune characteristics more akin to human immune responses: while specific pathogen-free mice and co-housed laboratory mice with pet-store mice were similarly susceptible to acute influenza infection, vaccinations in laboratory mice co-housed with pet-store mice dampened humoral and T-cell responses, leading to poor control upon challenge [86]. In addition, SA’s affinity for human-encoded immune factors could also contribute to discrepant results between laboratory animal and human hosts, indicating the limited translational potential of the current mouse models. Thus, humanized CD34^+^ (huCD34) and humanized PBMC (huPBMC) mouse models, which generate multi-lineage human immune cells that could interact with human tropic SA, could be an optimal platform to study SA vaccine efficacy. Likewise, animals transgenic for human immune factors known to interact with human-tropic SA factors [87,88], could serve as adjunctive tools for SA vaccine research. Human organoids also provide a means to test human T- and B-cell function, especially if the organoids are derived from the same human donors. Thus, short of a direct human challenge, models with human immune elements could provide insights into understanding the complex immune environment related to human vaccination.

Vaccine antigens are clearly of paramount importance in the development of SA vaccines. In our recent study, we aimed at characterizing the humoral imprints induced by primary SA infection, with the goal of identifying strategies to mitigate their negative impact on immunization [71]. Remarkably, we showed that the antibody titer and the protective function of the humoral imprint alone accurately predicted the efficacy of antigen-specific vaccines tested in mice [71]. Specifically, we demonstrated that immunodominant CWA antigens, as determined by the antibody titer post-primary SA infection, elicited non-protective humoral imprints, while subdominant CWA antigens that resulted in protective humoral imprints were effective in SA pre-infected mice. Active vaccination targeting toxins also induced protective imprints, although abundant anti-toxin Abs are found in many/most individuals on reaching adulthood, which begs the question of how much additional benefit could be derived from vaccinating individuals against selective toxins (e.g., α-toxin) after a threshold level of protective Abs has been reached. It is important to point out that the characterization of dominant versus subdominant CWA antigens is based on our murine model, and that there are inherent differences between humans and mice. For example, while MntC-specific antibody levels are robust and persistent after a single SA infection in C57BL/6 mice, they are modest in human sera [71]. Therefore, the in-depth screening of antigens is essential when selecting candidates for future clinical trials.

Since SA survival in diverse tissue depends on different virulence mechanisms, a multicomponent vaccine is thought to be necessary to induce significant anti-SA immunity. In addition, vaccine developers have gravitated towards multicomponent vaccines as a result of a lack of success of SA vaccine trials [89], with the rationale that the vaccine would be successful if one of the vaccine antigens is protective. IL-10, often associated with distinct types of SA infections, plays a role in suppressing the development of protective T-cell responses. SA infection has been demonstrated to induce IL-10-producing Tregs imprints both in humans and mice [39,69]. Based on our imprint hypothesis, non-protective SA antigens that induce IL-10 could induce the cross-suppression of otherwise protective vaccine responses. Hence, as a caution, more may not necessarily be better.

Choosing an appropriate adjuvant is of tantamount importance in driving an appropriate SA-directed immune response. Although Alum has historically been the favored prototype adjuvant, studies including our own have demonstrated its lack of efficiency in eliciting protective immunity in mice previously infected with SA. Notably, we have shown that SA infections interfere with the development of Th17 immune responses, suggesting that selecting an adjuvant capable of inducing potent Th17 immunity could offer substantial protection against SA [48]. Work by Montgomery and colleagues has revealed that a Th17-inducing CAF01 adjuvant can abrogate interference with an anti-toxin vaccination that is induced by prior SA infection [79]. A successful adjuvant strategy that similarly reverses the non-protective CWA vaccine would be a major step towards addressing the dilemma posed by immune imprinting. Pertinently, exploring the utility of Th17 adjuvants, such as CAF01, monophosphoryl lipid A [90], or STING-activating cyclic dinucleotides (CDNs) [91], alongside CWA antigens would be important as an approach to overcome the vaccine interference established by IL-10.

Clinical trial design likely also contributed to the failure of vaccines. For example, while the selection of patients at an elevated risk for infection (e.g., those undergoing cardiothoracic surgery) allows for a reduced trial size, sick or older populations likely have suboptimal opsonic functions or have abundant non-protective immune imprints that make vaccination less likely to be successful. Accordingly, the vaccination of infants could circumvent non-protective imprinting, although it is unclear how maternal anti-SA Abs that are transferred to the infants pre-birth and through breastfeeding would affect the SA vaccination of infants.

Since SA infection occurs at a relatively low frequency. The absence of established correlates of protection in humans poses a significant challenge in assessing vaccine efficacy in clinical trials. Thus, identifying reliable markers of protective immunity or correlates of protection will be critical for effective vaccine development. Building on insights gained from the study of vaccines in mice pre-exposed to SA, the level of IL-10 produced by anti-SA adaptive immune cells could prove to be a good indicator of vaccine efficacy, as could the use of the in vivo protection assay that assesses human anti-SA Ab function in mice [92]. Besides IL-10, research has shown that the basophil-derived Th2 cytokine, IL-4, promotes cutaneous SA infection by inhibiting IL-17A production by T cells [47]. Thus, assessing IL-4 production in conjunction with IL-10 could point to additional markers for predicting the efficacy of SA vaccines. A more detailed characterization of anti-SA Abs could yet reveal further structural information that serve as reliable and easily surveyable correlates for human vaccine studies. For practical purpose, T cells and Abs from Phase I vaccinated subjects could be evaluated for their protective function prior to advancement to costlier Phase II or III trials.

## 6. Conclusions

It is notable that some of the most difficult to develop antimicrobial vaccines to date (e.g., against tuberculosis, malaria, and SA) have been against pathogens that have forged a close relationship with humankind. The longstanding relationship with the human host has allowed pathogens to evolve effective ways to neutralize potent adaptive immune defenses. Therefore, it is not a surprise that some of the pathogen mechanisms that blunt host T- and B-cell responses also critically affect vaccination. For the pathobiont SA, the hijacking of host immunosuppressive IL-10 mechanism is an attractive hypothesis that explains the broad failure of staphylococcal vaccines in the context of immune imprinting. But much remains to be explored on how and the extent to which IL-10 undermines T- and B-cell vaccines, and what other factors are involved. These investigations could yet uncover additional bacterial or host targets to facilitate vaccine development. While the findings in mice provide a plausible explanation for why SA vaccines failed, more focus needs to be directed on exploring human data that directly inform on the mechanisms of failed human vaccines. Thus, generating better translational tools and directly assessing the validity of the hypothesis in clinical trials will be important going forward. In parallel, investigating human-relevant subdominant SA antigens and potent IL-17-inducing adjuvants should be a priority for successful SA vaccination in humans.
